# Breast cancer risk factors, survival and recurrence, and tumor molecular subtype: analysis of 3012 women from an indigenous Asian population

**DOI:** 10.1186/s13058-018-1033-8

**Published:** 2018-09-18

**Authors:** Mustapha Abubakar, Hyuna Sung, Devi BCR, Jennifer Guida, Tieng Swee Tang, Ruth M. Pfeiffer, Xiaohong R. Yang

**Affiliations:** 1 Integrative Tumor Epidemiology Branch, Division of Cancer Epidemiology and Genetics, National Cancer Institute (NCI), National Institutes of Health, 9609 Medical Center Drive, Rockville, MD 20850 USA; 2 Surveillance and Health Services Research, American Cancer Society, 250 Williams Street NW, Atlanta, GA 30303 USA; 3 Department of Radiotherapy, Oncology and Palliative Care, Sarawak General Hospital, Kuching, Sarawak Malaysia; 4 Division of Cancer Control & Population Sciences, National Cancer Institute, National Institutes of Health, Rockville, MD USA

**Keywords:** Breast cancer, Risk factors, Survival, Recurrence, Molecular subtype

## Abstract

**Background:**

Limited evidence, mostly from studies in Western populations, suggests that the prognostic effects of lifestyle-related risk factors may be molecular subtype-dependent. Here, we examined whether pre-diagnostic lifestyle-related risk factors for breast cancer are associated with clinical outcomes by molecular subtype among patients from an understudied Asian population.

**Methods:**

In this population-based case series, we evaluated breast cancer risk factors in relation to 10-year all-cause mortality (ACM) and 5-year recurrence by molecular subtype among 3012 women with invasive breast cancer in Sarawak, Malaysia. A total of 579 deaths and 314 recurrence events occurred during a median follow-up period of ~ 24 months. Subtypes (luminal A-like, luminal B-like, HER2-enriched, triple-negative) were defined using immunohistochemical markers for hormone receptors and human epidermal growth factor receptor 2 (HER2) in conjunction with histologic grade. Hazard ratios (HRs) and 95% confidence intervals (CIs) for the associations between risk factors and ACM/recurrence were estimated in subtype-specific Cox regression models.

**Results:**

We observed heterogeneity in the relationships between parity/breastfeeding, age at first full-term pregnancy (FFP), family history, body mass index (BMI), and tumor subtype (*p* value < 0.05). Among luminal A-like patients only, older age at menarche [HR (95% CI) _≥15 vs ≤ 12 years_ = 2.28 (1.05, 4.95)] and being underweight [HR_BMI < 18.5kg/m_^2^_vs. 18.5–24.9kg/m_^2^ = 3.46 (1.21, 9.89)] or overweight [HR_25–29.9kg/m_^2^_vs. 18.5–24.9kg/m_^2^_=_ 3.14 (1.04, 9.50)] were associated with adverse prognosis, while parity/breastfeeding [HR_breastfeeding vs nulliparity_ = 0.48 (0.27, 0.85)] and older age at FFP [HR _> 30 vs < 21 years_ = 0.20 (0.04, 0.90)] were associated with good prognosis. For these women, the addition of age at menarche, parity/breastfeeding, and BMI, provided significantly better fit to a prognostic model containing standard clinicopathological factors alone [LRχ^2^ (8*df*) = 21.78; *p* value = 0.005]. Overall, the results were similar in relation to recurrence.

**Conclusions:**

Our finding that breastfeeding and BMI were associated with prognosis only among women with luminal A-like breast cancer is consistent with those from previously published data in Western populations. Further prospective studies will be needed to clarify the role of lifestyle modification, especially changes in BMI, in improving clinical outcomes for women with luminal A-like breast cancer.

## Background

In addition to impacting incidence, lifestyle and environmental risk factors for breast cancer may influence disease progression. Several studies have previously evaluated this question, with mixed results. While some studies have documented older age at menarche [1–3], early age at first full-term pregnancy (FFP) [4, 5] and nulliparity [6, 7] to be associated with adverse prognosis in breast cancer patients, others have reported better prognosis in relation to these risk factors [8–12]. Discrepancies in reported associations may be explained by differences in study populations, risk factor distributions, and potential confounders, but could also be due to heterogeneity inherent in breast cancer.

Findings from expression profiling studies have been used to classify breast cancers into intrinsic subtypes (i.e. luminal A, luminal B, human epidermal growth factor receptor 2 (HER2)-enriched, basal-like, and normal-like subtypes), which were associated with different prognoses [13] and can be corroborated by immunohistochemical (IHC) markers for hormone receptors (i.e. estrogen receptor (ER), progesterone receptor [PR]) and HER2. Recently, proxies of the extent of tumor proliferation have been endorsed to refine subgroups that recapitulate the intrinsic subtypes more accurately than using hormone receptors and HER2 alone [14, 15]. Epidemiological studies have shown that associations between breast cancer risk factors vary by tumor subtypes. For example, parity and early age at FFP are associated with decreased risk of luminal breast tumors, but they do not protect and may even increase the risk for ER-negative or triple-negative breast cancers [16–19].

Three previous studies have evaluated the relationship between breast cancer risk factors and survival according to molecular subtype, one among women in Seoul, South Korea [20] and the other two involving analyses of US-based prospective breast cancer cohorts [21, 22]. Results from these studies suggest that the associations between late age at menarche [20], breastfeeding [21], high body mass index (BMI) [22] and survival after breast cancer might differ according to molecular subtype. However, findings from these studies are yet to be validated in independent populations and, to our knowledge, no study has specifically examined risk factors in relation to survival according to subtypes defined by the recent IHC classification scheme accounting for proliferation in an Asian population.

Despite racial and geographic variations in the incidence, presentation, and outcome of breast cancer; so far, most investigations on risk factors in relation to tumor subtypes and survival have been conducted in European populations. This analysis, therefore, aims to evaluate the association between breast cancer risk factors and tumor molecular subtypes, defined by hormone receptors and HER2 in conjunction with histologic grade; and to examine the relationship between risk factors and survival by molecular subtype among women in Sarawak, Malaysia.

## Methods

### Study population

Sarawak is a Malaysian state on Borneo with a multiethnic composition, comprising of native Borneo populations (51%), Chinese (25%) and Malays (24%) [23]. Overall, 3355 women with invasive breast cancer diagnosed and treated between 2003 and 2016 in the Department of Radiotherapy, Oncology, and Palliative Care, Sarawak General Hospital where ~ 93% of all breast cancer cases diagnosed in Sarawak are treated, were recruited for this study. Of these, 106 (~ 3%) did not participate by not filling the questionnaire leading to a participating rate of ~ 97%. Of the 3249 who participated, 168 (~ 5%) were lost to follow-up and 69 did not have complete information on ER, PR, HER2, and grade that is needed to generate breast cancer subtypes hence were excluded from further analysis. Ultimately, 3012 women representing ~ 90% of the original population were included in the current analysis. Information on lifestyle and environmental risk factors were obtained from questionnaires that were administered to participants at enrollment, which was approximately 4 weeks after diagnosis, while information on tumor characteristics was obtained from clinical records. Weight and height measurements were obtained in the clinic as part of the clinical workup for the calculation of chemotherapy doses. Recordings were performed by a trained member of staff using a weighing scale. Patients were given follow-up appointments to the clinic during which recurrence was evaluated and clinically confirmed. For those living in the outskirts of the city, if recurrence was suspected, the patients were referred to our clinic for further evaluation. Furthermore, a research assistant made regular calls to check the patient’s status, whether alive or dead. The current analysis included a follow-up period of 153 months (median follow-up = 24 months). Ethical approval for this project was provided by the Ethics Committee of the National Institutes of Health, Malaysia. This study did not involve the use of personal identifying information; hence, it was exempted from review by the National Institutes of Health (NIH) Office of Human Subject Research Protections [23].

### Breast cancer subtype definition

IHC staining for ER, PR, and HER2 was performed on formalin-fixed, paraffin-embedded tissue sections as has been previously described [24]. Molecular subtypes were defined using the St Gallen classification, proposed for the recapitulation of the intrinsic subtypes using IHC and proliferation markers [14, 15]. According to the St Gallen classification scheme, luminal breast cancers can be further distinguished into subgroups based on their level of proliferation (using KI67 or histologic grade) and hormone receptor expression patterns. Accordingly, luminal tumors that homogeneously express hormone receptors (i.e. ER+ and PR+) and low proliferation are classified as luminal A-like while those that heterogeneously express hormone receptors (i.e. ER^+^/PR^−^ or ER^−^/PR^+^) and/or those that homogeneously or heterogeneously express hormone receptors (i.e. ER+ and/or PR+) but are also high proliferating (high KI67 or grade 3) and/or HER2+ are classified as luminal B. In keeping with this definition, we utilized ER, PR, and HER2 in addition to histologic grade [25], to define subtypes as follows: Luminal A-like: ER^+^ and PR^+^, HER2^−^ and low grade (histologic grade 1 or 2); Luminal B-like: ER^+^ and/or PR^+^, HER2^−^ and high-grade (histologic grade 3) or ER^+^ and/or PR^+^, HER2^+^ (regardless of levels of histologic grade); HER2-enriched: ER^−^ and PR^−^ and HER2^+^; and triple-negative: ER^−^ and PR^−^ and HER2^−^.

### Statistical analysis

Frequency tables were used to assess the distribution of risk factors and clinicopathological characteristics among the different subtypes. The chi-square test was used to assess differences for categorical variables and the Kruskal-Wallis test was used for continuous variables.

We categorized risk factors based on what is the convention for each variable and in accordance with what has been published in large-scale studies of breast cancer [18, 26]. We categorized age at menarche [≤12 years (early menarche), 13, 14 and ≥ 15 years (late menarche)]; family history of breast cancer in a first-degree relative [yes and no]; age at FFP [< 21, 21–24.9, 25–30, > 30 years] and age at menopause [≤ 50 and > 50 years] similarly as in previously published articles [18, 26]. For BMI, we adopted the World Health Organization classification [< 18.5 kg/m^2^ (underweight); 18.5–24.9 kg/m^2^ (normal weight); 25–29.9 kg/m^2^ (overweight) and ≥ 30 kg/m^2^ (obese)]. To test for associations between risk factors and molecular subtypes, we constructed a polytomous logistic regression model with tumor subtype as the outcome (luminal A-like subtype as the reference category) and risk factors (age at menarche [≤ 12 years (early menarche, reference category), 13, 14, and ≥ 15 years (late menarche)], parity and breastfeeding [nulliparity (reference category), parity but no breastfeeding, parity and breastfeeding], age at FFP [< 21 (reference category), 21–24.9, 25–30, > 30 years], family history [yes and no (reference category)], and BMI [< 18.5 kg/m^2^ (underweight); 18.5–24.9 kg/m^2^ (normal weight, reference category); 25–29.9 kg/m^2^ (overweight) and ≥ 30 kg/m^2^ (obese)] as explanatory variables, with adjustment for age at diagnosis (< 35, 35–45, 45–55, 55–65, 65–75, > 75 years) and ethnicity (Chinese, Malay, Native).

The association between breast cancer subtypes and all-cause mortality/recurrence was determined using Kaplan-Meier survival curves and Cox-proportional hazards regression models, which included adjustments for standard prognostic parameters including age at diagnosis, ethnicity, BMI, histologic grade, TNM stage I–IV [i.e. size (T), nodal status (N) and metastasis (M)], systemic therapy (endocrine (tamoxifen or aromatase inhibitor (AI)) and chemotherapy), radiotherapy and surgery. Follow-up started at diagnosis of breast cancer and ended at time of event (recurrence/death) or censoring (end of follow-up or, for the recurrence analysis, also death). For all-cause mortality, we censored at 10 years because this is the threshold at which most breast cancers are, by convention, considered cured in the absence of recurrence or death. We adopted a two-step approach in our survival analyses. In the first step, each of the above risk factors was modeled separately in basic models adjusted for standard prognostic factors separately for each tumor subtype. To test for heterogeneity in risk factor and survival relationships by subtype, we included an interaction term between each risk factor and tumor subtype. Violation of the proportionality assumption of the hazard model was tested by modeling each risk factor as a time-varying covariate. In the second step, it was decided, a priori, that factors that were associated with survival with *P* <  0.1 in the basic model were to be mutually adjusted for in a multivariable model that included the standard prognostic factors mentioned above. Using likelihood ratio (LR) test, we compared this model with one containing only the clinicopathological factors. For sensitivity analysis, we conducted survival analysis for women stratified into two age groups (< 50 yrs and ≥ 50 yrs). Also, we performed additional sensitivity analysis by excluding women with stage IV disease from our multivariate analyses for both all-cause mortality and recurrence. Results were very similar from these sensitivity analyses as compared to analyses including all women and we therefore presented results from all patients. All analyses were two-sided and performed using Stata statistical software version 14.0 (StataCorp, College Station, TX, USA).

## Results

In total, our analysis included 3012 invasive breast cancer cases, with a total of 579 deaths in 10 years and 314 recurrence events in 5 years. The mean age at diagnosis was 52 years and mean BMI was 25 kg/m^2^. The majority of the patients were Chinese (48%) and had early-stage (I and II, 56%) and HR-positive (66%) tumors (Table 1). Of the 3012 patients, 1016 (34%) were luminal A-like, 989 (33%) were luminal B-like, 387 (13%) were HER2-enriched, and 620 (20%) were triple-negative, respectively.

**Table 1:** Distribution of risk factors and clinicopathological characteristics by tumor subtype

Characteristic	Overall (%)	A-like (*N* = 1016/34%)	%	B-like (*N* = 989/33%)	%	HER2-enriched (*N* = 387/13%)	%	Triple-neg. (*N* = 620/20%)	%	*P* value^a^
Age, yrs										
Mean (range)	51.6 (19, 91)	52.6 (24–90)		51.1 (19, 90)		51.6 (23, 91)		51.5 (21, 87)		
< 35	180 (5)	47	4.6	49	5.0	26	6.7	40	6.5	**0.01**
35–45	711 (21)	192	18.9	219	22.1	76	19.7	144	23.2	
45–55	1204 (36)	368	36.2	366	37.0	136	35.1	203	32.7	
55–65	806 (24)	241	23.7	246	24.9	105	27.1	143	23.1	
65–75	364 (11)	135	13.3	90	9.1	35	9.1	65	10.5	
> 75	90 (3)	33	3.3	19	1.9	9	2.3	25	4.0	
Ethnicity										
Chinese	1626 (48)	567	55.8	435	44	180	46.5	275	44.4	**< 0.0001**
Malay	801 (24)	204	20.1	263	26.6	104	26.9	155	25	
Native	928 (28)	245	24.1	291	29.4	103	26.6	190	30.6	
Menarche										
≤ 12 yrs	1105 (33)	344	34.1	326	33.2	118	30.8	190	30.9	0.20
13 yrs	1117 (34)	318	31.5	340	34.7	148	38.6	201	32.7	
14 yrs	548 (16)	163	16.2	167	17	60	15.7	111	18	
≥ 15 yrs	559 (17)	184	18.2	148	15.1	57	14.9	113	18.4	
Menopause										
≤ 50 yrs	428 (29)	146	32.7	128	29.7	38	23.2	87	31.6	0.14
> 50 yrs	1031 (71)	301	67.3	303	70.3	126	76.8	188	68.4	
Parity										
Nulliparous	745 (22)	242	23.8	224	22.6	77	19.9	128	20.6	0.29
Parous	2601 (78)	774	76.2	765	77.4	310	80.1	492	79.4	
Age at FFP^b^, yrs										
< 21	466 (18)	116	15	158	20.7	54	17.4	95	19.3	**0.02**
21–24.9	1011 (39)	284	36.7	287	37.5	129	41.6	199	40.5	
25–30	864 (33)	275	35.5	248	32.4	103	33.2	154	31.4	
> 30	259 (10)	99	12.8	72	9.4	24	7.7	43	8.7	
Breastfeeding										
No	377 (14)	133	17.2	103	13.5	31	10	64	13	**0.01**
Yes	2224 (86)	641	82.9	662	86.5	279	90	428	87	
Breastfeeding duration										
< 6 months	927 (52)	285	54.2	280	51.8	111	49.6	175	51.3	0.40
6–10 months	374 (21)	112	21.3	122	22.6	48	21.4	62	18.2	
> 10 months	489 (27)	129	24.5	138	25.6	65	29.0	104	30.5	
BMI, kg/m^2^										
< 18.5	1253 (39)	373	37.8	336	35.1	163	43.7	240	40.2	**0.03**
18.5–24.9	565 (17)	160	16.2	172	18	68	18.2	114	19.1	
25–29.9	998 (31)	317	32.2	301	31.4	104	27.9	177	29.6	
≥ 30	433 (13)	136	13.8	148	15.5	38	10.2	66	11.1	
Family history										
No	2835 (86)	840	84	854	87.8	338	88	516	84.9	**0.05**
Yes	468 (14)	160	16	119	12.2	46	12	92	15.1	
Histological grade										
Well diff.	365 (11)	212	20.9	67	6.8	10	2.6	27	4.4	**< 0.0001**
Moderately diff.	1790 (55)	781	76.9	442	44.7	180	46.5	238	38.4	
Poorly diff.	1123 (34)	–	–	473	47.8	192	49.6	344	55.5	
Stage										
I	454 (14)	222	22.1	100	10.3	34	8.9	61	10	**< 0.0001**
II	1353 (42)	444	44.2	395	40.6	136	35.6	249	40.9	
III	1005 (31)	241	24	330	33.9	150	39.3	203	33.3	
IV	421 (13)	78	7.8	139	14.3	57	14.9	91	14.9	
Tumor size										
< 2 cm	2137 (64)	761	75.6	620	63.3	210	54.7	353	57.8	**< 0.0001**
2–5 cm	522 (16)	121	12	150	15.3	83	21.6	104	17	
> 5 cm	660 (20)	125	12.4	209	21.4	91	23.7	154	25.2	
Node status										
0	1517 (46)	566	55.7	368	37.2	146	37.7	272	43.9	**< 0.0001**
1	922 (28)	249	24.5	310	31.3	95	24.5	163	26.3	
2	480 (15)	120	11.8	156	15.8	70	18.1	94	15.2	
≥ 3	375 (11)	72	7.1	139	14.1	68	17.6	75	12.1	
Endocrine										
None	1248 (40)	48	5.1	126	14.2	357	97.5	584	97.2	**< 0.0001**
Tamoxifen	1456 (47)	687	72.9	591	66.7	7	1.9	15	2.5	
Aromatase Inhibitor	395 (13)	207	22.0	169	19.1	2	0.6	2	0.3	
Chemotherapy										
No	799 (24)	371	37.3	177	18.3	62	16.4	95	15.5	**< 0.0001**
Yes	2483 (76)	625	62.7	789	81.7	317	83.6	519	84.5	
Surgery										
No	389 (12)	80	10.2	106	15.8	53	19.4	87	16.3	**< 0.0001**
Yes	2841 (88)	913	89.8	833	84.2	312	80.6	519	83.7	
Radiotherapy										
No	800 (26)	261	27.7	218	24.6	79	23.9	149	26.4	**0.01**
Yes	2235 (74)	683	72.3	669	75.4	252	76.1	416	73.6	

Breast cancer subtypes were defined based on 2013 St Gallen criteria by using hormone receptor (ER and PR) and HER2 in conjunction with histologic grade. In bold are statistically significant *P* values (< 0.05)

^a^*P* values are for chi-square tests

^b^*FFP* first full-term pregnancy

### Distribution of risk factors and clinicopathological characteristics by tumor subtype

As shown in Table 1, women with the luminal A-like subtype were slightly older than those with other subtypes. The distributions of ethnicity (*P* value < 0.001), age at FFP (*P* value = 0.019), breastfeeding practices (*P* value = 0.01), family history (*P* value = 0.05) and BMI (*P* value = 0.03) differed by subtype. No differences were observed in the distributions of age at menarche, age at menopause and parity according to subtype. The frequencies of all clinicopathological parameters differed by subtype, with low-grade, small, early-stage and node-negative tumors being more frequent for the luminal A-like subtype (Table 1).

Table 2 shows the associations between examined risk factors and molecular subtype in the multivariable polytomous regression model. Compared with women with the luminal A-like subtype, women with the luminal B-like, HER2-enriched and triple-negative tumors were significantly more likely to be Malay and Native than Chinese. Furthermore, women with other tumor subtypes were more likely to be parous and have breastfed [odds ratio (OR) (95% CI) parity and breastfeeding vs nulliparity = 1.44 (1.05, 1.98); 1.64 (1.06, 2.25); and 1.54 (1.07, 2.22) for luminal B, HER2-enriched and triple-negative subtypes, respectively] and less likely to experience their FFP after the age of 30 years [OR (95% CI) > 30 years vs < 21 years = 0.63 (0.42, 0.94); 0.57 (0.32, 1.02); and 0.58 (0.36, 0.93) for luminal B, HER2-enriched and triple-negative subtypes, respectively] than those with the luminal A-like subtype. Women with HER2-enriched [OR (95% CI) BMI > 30 kg/m^2^ vs 18.5–24.9 kg/m^2^ = 0.55 (0.34, 0.89); *P* value = 0.02] and triple-negative [OR (95% CI) BMI > 30 kg/m^2^ vs 18.5–24.9 kg/m^2^ = 0.59 (0.40, 0.88); *P* value = 0.01] tumors were significantly less likely to be obese than those with the luminal A-like subtype.

**Table 2:** OR and 95% CI from a polytomous logistic regression model testing the associations between breast cancer risk factors and tumor molecular subtype

Risk factor	Subtype						
	A-like (comparison group)	B-like		HER2-enriched		Triple-negative	
	N	N	OR^a^ (95% CI)	N	OR (95% CI)	N	OR (95% CI)
Ethnicity							
Chinese	567	435	1.00 (reference)	180	1.00 (reference)	275	1.00 (reference)
Malay	204	263	**1.50 (1.18, 1.90)**	104	**1.54 (1.13, 2.11)**	155	**1.51 (1.14, 1.98)**
Native	245	291	**1.39 (1.10, 1.76)**	103	1.21 (0.88, 1.66)	190	**1.50 (1.15, 1.95)**
*P* value			**0.001**		0.10		**< 0.001**
Menarche							
≤ 12 yrs	344	326	1.00 (reference)	118	1.00 (reference)	190	1.00 (reference)
13 yrs	318	340	1.14 (0.91, 1.43)	148	1.31 (0.98, 1.77)	201	1.15 (0.88, 1.49)
14 yrs	163	167	1.04 (0.79, 1.38)	60	1.02 (0.70, 1.48)	111	1.18 (0.86, 1.61)
≥ 15 yrs	184	148	0.83 (0.63, 1.10)	57	0.87 (0.59, 1.28)	113	1.10 (0.80, 1.50)
*P* value			0.43		0.51		0.31
Parity and BF^b^							
Nulliparous	242	224	1.00 (reference)	77	1.00 (reference)	128	1.00 (reference)
Parous and No BF	133	103	1.26 (0.84, 1.90)	31	1.07 (0.76, 1.52)	64	1.25 (0.77, 2.00)
Parous and BF	641	662	**1.44 (1.05, 1.98)**	279	**1.64 (1.06, 2.55)**	428	**1.54 (1.07, 2.22)**
*P* value			0.17		**0.005**		0.12
Age at FFP^c^							
< 21 yrs	116	158	1.00 (reference)	54	1.00 (reference)	95	1.00 (reference)
21–24.9 yrs	284	287	0.78 (0.57, 1.05)	129	1.01 (0.67, 1.51)	199	0.90 (0.64, 1.26)
25–30 yrs	275	248	0.77 (0.57, 1.06)	103	0.91 (0.60, 1.38)	154	0.77 (0.54, 1.09)
> 30 yrs	99	72	**0.63 (0.42, 0.94)**	24	0.57 (0.32, 1.02)	43	**0.58 (0.36, 0.93)**
*P* value			0.35		0.06		0.38
Family history							
No	840	854	1.00 (reference)	338	1.00 (reference)	516	1.00 (reference)
Yes	160	119	**0.72 (0.55, 0.94)**	46	0.71 (0.49, 1.02)	92	0.92 (0.69, 1.23)
*P* value			**0.02**		**0.05**		0.62
BMI, kg/m^2^							
18.5–24.9	160	172	1.00 (reference)	68	1.00 (reference)	114	1.00 (reference)
< 18.5	373	336	0.89 (0.69, 1.17)	163	1.07 (0.76, 1.51)	240	0.96 (0.72, 1.29)
*P* value			0.38		0.65		0.76
25–29.9	317	301	0.86 (0.65, 1.12)	104	0.74 (0.51, 1.06)	177	0.75 (0.55, 1.02)
*P* value			0.27		0.13		0.08
≥ 30	136	148	0.90 (0.65, 1.24)	38	**0.55 (0.34, 0.89)**	66	**0.59 (0.40, 0.88)**
*P* value			0.62		**0.02**		**0.01**

Statistically significant (*P* value < 0.05) estimates are indicated in bold

^a^OR and corresponding estimates are from a single polytomous logistic regression model that was mutually adjusted for ethnicity, menarche, parity and breastfeeding, age at FFP, family history, BMI and age

^b^*BF* breastfeeding

^c^*FFP* first full-term pregnancy

### Breast cancer risk factors in relation to all-cause mortality and recurrence by subtype

**Fig. 1:**
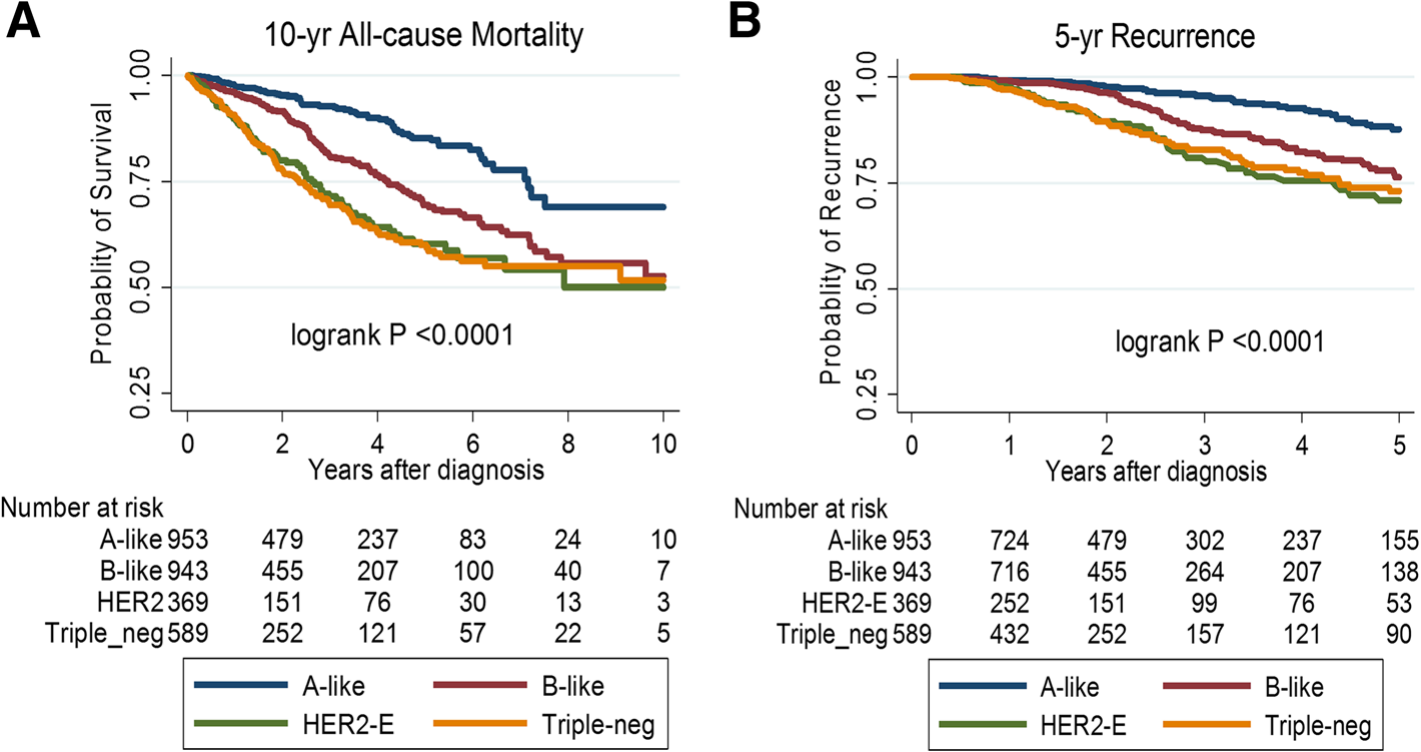
Kaplan-Meier curves for the associations between breast cancer molecular subtypes and (**a**) 10-year all-cause mortality (**b**) 5-year recurrence-free survival among 3012 women diagnosed and treated in Sarawak General Hospital, Sarawak Malaysia

Overall, all-cause mortality and recurrence differed significantly by tumor subtype. In general, women with luminal A-like tumors had better survival outcomes than those with the other subtypes (Fig. 1). As shown in Table 3, in basic models for each risk factor (with adjustment for standard prognostic factors in addition to age, ethnicity and BMI), later age at menarche, parity/breastfeeding, and being underweight were significantly associated with 10-year all-cause mortality in the luminal A-like but not any of the other subtypes. Also, later age at FFP showed a suggestive association with mortality in luminal A-like patients (*P* trend = 0.08) but not the other subtypes. Results were similar in basic models for recurrence (Table 4).

**Table 3:** HR and 95% CI for the associations between risk factors and 10-year all-cause mortality by tumor molecular subtype

Risk factor^1a^	10-year all-cause mortality								P het^d^
	A-like		B-like		HER2-enriched		Triple-negative		
	N/events	HR (95% CI)	N/events	HR (95% CI)	N/events	HR (95% CI)	N/events	HR (95% CI)	
Ethnicity									
Chinese	567/39	1.00 (reference)	435/41	1.00 (reference)	180/38	1.00 (reference)	275/57	1.00 (reference)	0.81
Malay	204/17	1.23 (0.65, 2.34)	263/70	**2.32 (1.49, 3.60)**	104/29	1.11 (0.63, 1.96)	155/54	1.22 (0.79, 1.89)	
Native	245/19	1.04 (0.52, 2.05)	291/39	1.68 (1.02, 2.77)	103/20	0.93 (0.48, 1.79)	190/41	1.06 (0.67, 1.67)	
*P* value		0.80		**0.03**		0.87		0.77	
Menarche									
≤ 12 yrs	344/14	1.00 (reference)	326/52	1.00 (reference)	118/23	1.00 (reference)	190/46	1.00 (reference)	0.06
13 yrs	318/26	1.53 (0.76, 3.11)	340/44	0.88 (0.55, 1.40)	148/29	0.84 (0.45, 1.57)	201/46	1.21 (0.76, 1.92)	
14 yrs	163/11	1.25 (0.54, 2.91)	167/27	0.86 (0.50, 1.48)	60/22	0.92 (0.47, 1.80)	111/26	1.05 (0.61, 1.80)	
≥ 15 yrs	184/23	**2.25 (1.06, 4.78)**	148/27	0.84 (0.49, 1.42)	57/11	0.49 (0.21, 1.11)	113/31	0.89 (0.52, 1.55)	
*P* value		**0.06**		0.49		0.15		0.66	
Parity and BF^b^									
Nulliparous	242/24	1.00 (reference)	224/36	1.00 (reference)	77/18	1.00 (reference)	128/40	1.00 (reference)	0.28
Parous and No BF	133/10	0.59 (0.27, 1.32)	103/12	0.81 (0.37, 1.75)	31/8	0.87 (0.34, 2.25)	64/20	1.02 (0.52, 1.96)	
Parous and BF	641/41	**0.46 (0.26, 0.81)**	662/102	0.99 (0.64, 1.56)	279/61	0.61 (0.33, 1.14)	428/92	0.86 (0.55, 1.34)	
*P* value		**0.009**		0.91		0.11		0.48	
Age at FFP^c^									
< 21 yrs	116/10	1.00 (reference)	158/32	1.00 (reference)	54/13	1.00 (reference)	95/19	1.00 (reference)	0.22
21–24.9 yrs	284/14	0.99 (0.38, 2.55)	287/33	1.30 (0.75, 2.25)	129/28	0.83 (0.37, 1.84)	199/42	0.71 (0.39, 1.29)	
25–30 yrs	275/23	0.85 (0.41, 1.73)	248/39	1.14 (0.69, 1.90)	103/22	0.77 (0.40, 1.49)	154/41	0.98 (0.60, 1.60)	
> 30 yrs	99/4	0.36 (0.10, 1.29)	72/10	1.34 (0.64, 2.81)	24/6	0.78 (0.25, 2.46)	43/9	0.53 (0.23, 1.24)	
*P* value		**0.08**		0.92		0.15		0.58	
Family history									
No	840/56	1.00 (reference)	854/131	1.00 (reference)	338/73	1.00 (reference)	516/122	1.00 (reference)	0.25
Yes	160/17	1.70 (0.93, 3.08)	119/17	1.05 (0.59, 1.88)	46/13	1.47 (0.76, 2.82)	92/22	0.77 (0.46, 1.27)	
*P* value		**0.08**		0.86					
BMI, kg/m^2^									
18.5–24.9	160/6	1.00 (reference)	172/25	1.00 (reference)	68/15	1.00 (reference)	114/25	1.00 (reference)	0.80
< 18.5	373/38	**3.42 (1.20, 9.71)**	336/43	1.06 (0.62, 1.82)	163/37	0.83 (0.44, 1.57)	240/63	1.15 (0.70, 1.91)	
*P* value		**0.02**		0.83		0.58		0.56	
25–30	317/21	2.88 (0.97, 8.59)	301/49	1.30 (0.77, 2.20)	104/19	0.80 (0.39, 1.64)	177/40	0.92 (0.54, 1.57)	
*P* value		**0.06**		0.32		0.54		0.77	
> 30	136/7	1.30 (0.37, 4.52)	148/23	1.12 (0.61, 2.05)	38/10	1.23 (0.52, 2.90)	66/18	1.21 (0.63, 2.32)	
*P* value		0.68		0.71		0.63		0.57	

In bold are variables which met our criteria (*P* value < 0.1) for inclusion in multivariate models

^a^Each risk factor was adjusted for age, ethnicity, BMI, tumor stage, histologic grade, surgery, systemic therapy (endocrine (tamoxifen or AI versus none), chemotherapy (any regimen versus none)) and radiotherapy (received versus none)

^b^*BF* breastfeeding

^c^*FFP* first full-term pregnancy

^d^*P* value for heterogeneity (P-het) of HR estimates according to molecular subtypes

**Table 4:** HR and 95% CI for the associations between risk factors and 5-year recurrence by tumor molecular subtype

Risk factor^a^	Recurrence after 5 years								P-het^d^
	A-like		B-like		HER2-enriched		Triple-negative		
	N/events	HR (95% CI)	N/events	HR (95% CI)	N/events	HR (95% CI)	N/events	HR (95% CI)	
Ethnicity									
Chinese	567/34	1.00 (reference)	435/49	1.00 (reference)	180/30	1.00 (reference)	275/38	1.00 (reference)	0.96
Malay	204/8	1.01 (0.39, 2.60)	263/30	0.81 (0.45, 1.45)	104/17	0.69 (0.31, 1.51)	155/24	1.11 (0.61, 2.02)	
Native	245/16	1.17 (0.49, 2.79)	291/22	0.84 (0.46, 1.55)	103/10	0.47 (0.18, 1.23)	190/22	0.67 (0.34, 1.34)	
*P* value		0.75		0.55		0.11		0.32	
Menarche									
≤ 12 yrs	344/13	1.00 (reference)	326/38	1.00 (reference)	118/14	1.00 (reference)	190/34	1.00 (reference)	0.07
13 yrs	318/17	1.55 (0.57, 4.23)	340/31	1.03 (0.58, 1.82)	148/21	1.52 (0.69, 3.33)	201/24	0.72 (0.38, 1.37)	
14 yrs	163/14	2.51 (0.86, 7.33)	167/21	0.81 (0.40, 1.64)	60/9	0.56 (0.16, 1.92)	111/13	0.65 (0.30, 1.43)	
≥ 15 yrs	184/13	**3.26 (1.08, 9.92)**	148/11	0.77 (0.35, 1.68)	57/12	1.11 (0.38, 3.28)	113/12	0.61 (0.27, 1.36)	
*P* value		**0.02**		0.41		0.81		0.18	
Parity and BF^c^									
Nulliparous	242/19	1.00 (reference)	224/16	1.00 (reference)	77/12	1.00 (reference)	128/19	1.00 (reference)	0.56
Parous and No BF	133/7	0.55 (0.20, 1.50)	103/14	1.86 (0.76, 4.56)	31/7	1.35 (0.39, 4.64)	64/17	1.57 (0.67, 3.66)	
Parous and BF	641/32	**0.27 (0.12, 0.58)**	662/71	1.63 (0.81, 3.28)	279/38	0.58 (0.25, 1.34)	428/48	0.77 (0.39, 1.52)	
*P* value		**0.001**		0.23		0.15		0.28	
Age at FFP^d^									
< 25	400/13	1.00 (reference)	445/44	1.00 (reference)	183/30	1.00 (reference)	294/41	1.00 (reference)	0.52
≥ 25	374/26	1.69 (0.66, 4.34)	320/41	1.32 (0.78, 2.22)	127/15	0.50 (0.21, 1.16)	197/23	0.75 (0.41, 1.39)	
*P* value		0.27		0.28		0.10		0.36	
Family history									
No	840/42	1.00 (reference)	854/85	1.00 (reference)	338/49	1.00 (reference)	516/66	1.00 (reference)	0.94
Yes	160/13	0.77 (0.29, 2.03)	119/14	1.17 (0.60, 2.28)	46/7	1.24 (0.45, 3.35)	92/12	0.85 (0.43, 1.70)	
*P* value		0.60		0.64		0.67			
BMI, kg/m^2^									
18.5–24.9	160/6	1.00 (referent)	172/14	1.00 (referent)	68/11	1.00 (referent)	114/16	1.00 (referent)	0.98
< 18.5	373/30	3.97 (0.91, 17.34)	336/29	1.37 (0.62, 3.03)	163/21	0.50 (0.20, 1.27)	240/30	1.01 (0.51, 2.01)	
*P* value		**0.06**		0.43		0.15		0.97	
25–30	317/16	3.40 (0.75, 15.42)	301/37	2.02 (0.95, 4.29)	104/15	0.70 (0.27, 1.81)	177/24	0.83 (0.39, 1.73)	
*P* value		0.11		**0.06**		0.46		0.62	
> 30	136/5	1.65 (0.27, 10.15)	148/15	1.28 (0.53, 3.12)	38/7	1.41 (0.46, 4.27)	66/13	0.89 (0.35, 2.25)	
*P* value		0.59		0.58		0.55		0.81	

In bold are variables which met our criteria (*P*-value < 0.1) for inclusion in multivariate models

^a^Each risk factor was adjusted for age, ethnicity, BMI, tumor stage, histologic grade, surgery, systemic therapy (endocrine (tamoxifen or AI versus none), chemotherapy (any regimen versus none)) and radiotherapy (received versus none)

^b^*BF* breastfeeding

^c^*FFP* first full-term pregnancy. Due to sample size considerations age at FFP was dichotomized

^d^*P* value for heterogeneity (P-het) of HR estimates according to molecular subtypes

**Fig. 2:**
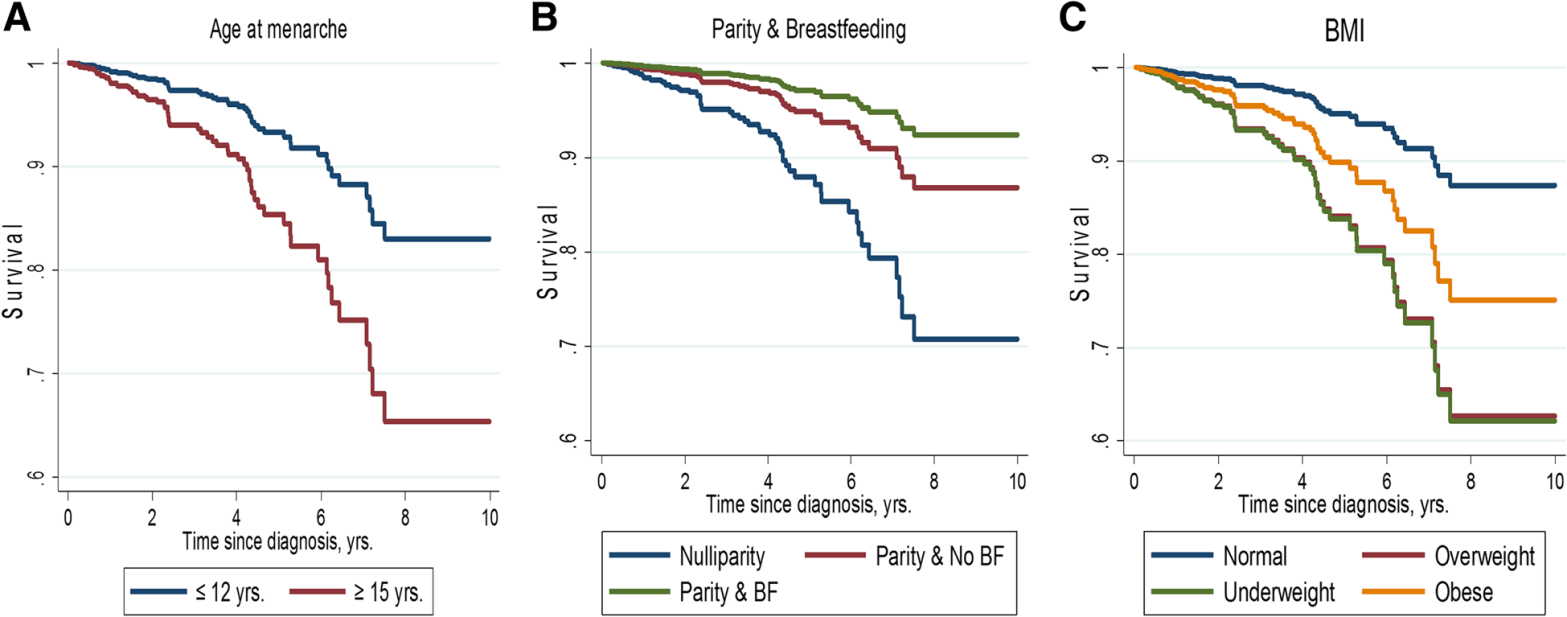
Survival curves for the multivariate association between (**a**) age at menarche, (**b**) parity and breastfeeding and (**c**) BMI and 10-year all-cause mortality among 1016 women with the luminal A-like subtype of breast cancer

In the multivariable model with the mutual adjustment for ethnicity, menarche, parity/breastfeeding, age at FFP, family history, and BMI in addition to standard clinicopathological factors and treatment variables, increasing age at menarche [hazard ratio (HR) (95% confidence interval (CI) ≥15 years vs ≤ 12 years = 2.28 (1.05, 4.95); *P* value for trend (*P* trend) = 0.06]; parity/breastfeeding [HR (95% CI) vs nulliparity = 0.48 (0.27, 0.85); *P* trend = 0.01]; older age at FFP [HR (95% CI) > 30 vs < 21 years = 0.20 (0.04, 0.90); *P* trend = 0.06]; and being underweight [HR (95% CI) vs normal weight = 3.46 (1.21, 9.89); *P* value = 0.02] or overweight [HR (95% CI) vs normal weight = 3.14 (1.04, 9.50); *P* value = 0.04] remained significantly associated with 10-year all-cause mortality in women with the luminal A-like subtype (Table 5 and Fig. 2). For these women, the addition of age at menarche, parity/breastfeeding, and BMI, provided significantly better fit to a model containing clinicopathological factors alone [LRχ^2^ (8*df*) = 21.78; *P* value = 0.005]. In general, the results were consistent in relation to recurrence (Table 5).

**Table 5:** Multivariate HR and 95% CI for the association between breast cancer risk factors and 10-year all-cause mortality and 5-year recurrence among women with luminal A-like subtype breast cancer

Risk factor	10-year all-cause mortality		5-year recurrence	
	N/events	HR^1^ (95% CI)	N/events	HR^a^ (95% CI)
Ethnicity				
Chinese	539/38	1.00 (reference)	399/21	1.00 (reference)
Malay	194/16	1.14 (0.59, 2.20)	154/7	0.81 (0.29, 2.27)
Native	235/17	0.87 (0.41, 1.85)	190/11	0.90 (0.35, 2.29)
*P* value		0.95		0.74
Menarche				
≤ 12 yrs	338/13	1.00 (reference)	260/8	1.00 (reference)
13 yrs	302/25	1.45 (0.70, 3.01)	235/13	1.69 (0.59, 4.81)
14 yrs	156/10	1.23 (0.52, 2.92)	119/8	2.64 (0.87, 7.99)
≥ 15 yrs	172/23	**2.28 (1.05, 4.95)**	129/10	**3.52 (1.10, 11.23)**
*P* value		0.06		**0.02**
Parity and BF^b^				
Nulliparous	229/23	1.00 (reference)	180/15	1.00 (reference)
Parous and No BF	127/9	0.61 (0.26, 1.42)	87/5	0.53 (0.17. 1.59)
Parous and BF	612/39	**0.48 (0.27, 0.85)**	476/19	**0.28 (0.13, 0.64)**
*P* value		**0.01**		**0.002**
Age at FFP^c^				
< 21 yrs	111/9	1.00 (reference)	85/4	1.00 (reference)
21–24.9 yrs	276/14	0.85 (0.31, 2.31)	220/4	0.40 (0.06, 2.28)
25–30 yrs	262/22	0.71 (0.27, 1.82)	201/17	1.26 (0.30, 5.22)
> 30 yrs	95/4	**0.20 (0.04, 0.90)**	73/1	0.26 (0.02, 3.09)
*P* value		0.06		0.95
Family history				
No	804/53	1.00 (reference)	629/31	1.00 (referent)
Yes	155/17	1.68 (0.91, 3.10)	122/7	0.75 (0.28, 2.02)
*P* value		0.10		0.57
BMI				
18.5–24.9 kg/m^2^	160/6	1.00 (reference)	117/2	1.00 (reference)
< 18.5 kg/m^2^	365/37	**3.46 (1.21, 9.89)**	285/22	4.31 (0.94, 19.83)
*P* value		**0.02**		0.06
25–29.9 kg/m^2^	310/21	**3.14 (1.04, 9.50)**	235/12	**4.74 (1.00, 22.64)**
*P* value		**0.04**		**0.05**
≥ 30 kg/m^2^	133/7	1.38 (0.39, 4.86)	106/3	2.16 (0.34, 13.78)
*P* value		0.61		0.41

In bold are statistically significant estimates (*P* value < 0.05)

^a^HR was mutually adjusted for ethnicity, menarche, parity and breastfeeding, age at FFP, family history, BMI, age, TNM stage, histologic grade, surgery, systemic therapy (endocrine (tamoxifen or AI versus none), chemotherapy (any regimen vs and radiotherapy (received versus none)

^b^*BF* breastfeeding

^c^*FFP* first full-term pregnancy

When we examined the association between duration of breastfeeding and all-cause-mortality/recurrence for luminal A-like cases with complete information on breastfeeding duration (*N* = 719), we observed an inverse association between each breastfeeding duration category and all-cause mortality [HR (95% CI) vs nulliparity = 0.37 (0.18, 0.85), 0.86 (0.35, 2.11), 0.53 (0.24, 1.17) for < 6, 6–10, and > 10 months, respectively (P trend = 0.38)] and recurrence [HR (95% CI) vs nulliparity = 0.47 (0.19, 1.16), 0.73 (0.23, 2.37), 0.05 (0.01, 0.42) for < 6, 6–10, and > 10 months, respectively (*P* trend = 0.002)]. Among women who breastfed, all-cause mortality did not significantly vary by breastfeeding durations (comparing > 10 months to < 6 months, *P* value = 0.38) but women who breastfed for > 10 months tended to have better recurrence outcomes [HR (95% CI) vs < 6 months = 0.11 (0.01, 0.93); *P* value = 0.04].

## Discussion

In this study involving over 3000 invasive breast cancer cases from a population-based case series in Sarawak, Malaysia, with detailed demographic, risk factor, pathology, and follow-up data, we investigated several established breast cancer risk factors in relation to tumor subtypes and patient outcomes. We found differences in the prevalence of parity and breastfeeding, age at FFP, family history of breast cancer and obesity across different breast tumor subtypes. In general, traditional breast cancer risk factors (older age at FFP, higher BMI, lower parity, lack of breastfeeding) seem to show higher frequencies among women with the luminal A-like subtype compared with women with other subtypes. Further, we found that age at menarche, breastfeeding, and BMI were independent prognostic factors for both overall mortality and breast cancer recurrence but only for women with the luminal A-like subtype, which had better survival and recurrence outcomes than the other subtypes.

Our findings that nulliparity and older age at FFP were more prevalent in luminal A-like patients are consistent with those reported in studies in Western countries [16, 27]. However, unlike the observation of higher BMI and shorter breastfeeding duration in triple-negative patients among Western, especially African American, women [18, 19, 28, 29], we found lower frequencies of obesity and breastfeeding among HR^−^ (HER2-enriched and triple-negative) than luminal A-like tumors, which may be reflective of population/ethnic differences. In line with this hypothesis, a previous study conducted in South Korea [30] also showed a higher frequency of breastfeeding among women with luminal B or HER2-enriched than those with luminal A disease. In another study involving 730 Mexican women with breast cancer, Martinez and colleagues [31] reported the prevalence of breastfeeding to be higher among women with triple-negative than luminal A tumors. Similarly, results from a multiethnic study showed an inverse association between triple-negative tumors and breastfeeding in White, Hispanic and African American but, notably, not in Asian women, for whom breastfeeding for > 2 months was associated with an 86% increased likelihood of triple-negative tumors [32].

The prevalence of obesity is still much lower in most Asian populations compared with other race/ethnicity groups. In contrast to the reduced breast cancer risk associated with higher BMI among premenopausal Western women, obesity is associated with increased risk for both premenopausal and postmenopausal Asian women [33–35]. The heterogeneity of obesity by tumor subtype among Asian cases remains unclear. Results from both our study and the South Korean study [30] suggest that obesity was less frequent among patients with HER2-enriched tumors. In combination with our finding that women with the HER2-enriched subtype were more likely to be parous and to have breastfed than women with the luminal A-like subtype, our data suggest that these factors (parity, breastfeeding, and low BMI) may not protect against HER2-enriched breast cancers. The decreasing prevalence of these factors associated with the adoption of westernized lifestyles may, therefore, not affect the incidence of this subtype, which is known to be more prevalent among Asian women [36, 37]. More research to understand the risk factors associated with the HER2-enriched subtype is warranted.

Most epidemiological studies considering the prognostic significance of age at menarche in breast cancer have treated the disease as a homogeneous entity, and results from these studies are largely conflicting [1–3, 8, 38]. We found that older age at menarche was associated with worse prognosis but only among women with the luminal A-like subtype. This finding is consistent with results from a previous study of women in East Asia [20] that also evaluated relationships between risk factors and survival according to subtypes. It is unclear why late menarche and younger age at FFP lead to worse survival outcomes in women with the luminal A-like subtype since they are well known protective factors in terms of breast cancer risk. One possibility is that because early menarche and late age at FFP increase breast cancer risk through prolonged and sustained exposure of the mammary epithelium to the mitogenic effects of reproductive hormones [39, 40], these factors predispose more strongly to HR^+^ tumors which have better prognosis than HR^−^ tumors [18, 41]. Although this association was confined to the luminal A-like subtype, which, by definition, is HR^+^, expression of hormone receptors in tumors occurs in a spectrum. Whereas some tumors have very high expression levels, others have lower levels despite crossing the threshold for consideration as HR^+^. Due to differences in cumulative lifetime exposure to endogenous estrogens, luminal A-like tumors occurring among women with late menarche may have lower levels of hormone receptor expression, hence worse survival/recurrence outcomes, than those occurring among women with early menarche. Indeed, this is consistent with the finding by Song et al. that longer duration of endogenous estrogen exposure was associated with better survival [30]. On the other hand, late menarche and early age at FFP may be indicative of lower socioeconomic status (SES) which, in turn, may be reflective of less exposure to “westernized” environments/lifestyles. Nonetheless, adjustment of known surrogates for SES did not change the associations between these factors and survival. That late age at menarche and early age at FFP were not associated with survival/recurrence in the other subtypes may be due to the masking effect of other more aggressive tumor features, which are inherent in these subtypes.

Our findings of breastfeeding being associated with better outcomes are generally consistent with previous reports [2, 12, 42]. A distinctive feature of luminal A-like tumors defined in our analysis is the low levels of proliferation, indicated by low histologic grade. Findings from one previous study showed that the protective effect of breastfeeding on breast cancer mortality was stronger for tumors with low expression of proliferation genes [21]. In our study, breastfeeding was associated with a preponderance of lobular carcinoma and small size tumors, both of which are highly correlated with low levels of proliferation [43–45].

Results from several studies, summarized in two comprehensive reviews and meta-analyses [46, 47], are supportive of the prognostic value of BMI in breast cancer. The association between BMI and survival after breast cancer is thought to be U-shaped [47–49], with underweight and overweight/obese women more likely to suffer worse survival outcomes than their normal weight counterparts. In our study, being underweight and overweight, but not obese, were suggestively associated with worse prognosis, but this might be due to the low frequency of obesity in this population (13%). Compared with overweight/obesity, the effect of underweight in breast cancer survival is less well-studied. Overall, our finding of an association between underweight and worse survival outcomes in breast cancer is in line with those of several other studies involving Asian populations [49–52]. Whilst insulin resistance, chronic inflammation, and altered adipokine and cytokine production have been proposed to underlie the obesity-cancer link [53], the precise mechanisms underpinning the relationship between underweight BMI and disease progression are not well understood. Chronic, pre-diagnostic, malnutrition may contribute to weight loss in cancer patients and may independently influence outcomes in the disease. However, when we examined the impact of indicators of socioeconomic deprivation, as surrogates for chronic malnutrition, our estimates remained unchanged.

Strengths of this study include: a population-based breast cancer case series in an understudied Asian population and the collection of detailed questionnaire information, which allowed us to account for various confounding variables including sociodemographic factors. Several limitations of this study should be noted. First, we did not include controls in our study and therefore our case-case comparisons could not be translated into relative risk estimates. Second, relatively small sample sizes for some of the subtypes may have affected our power to detect significant associations. Third, data on the specific cause of death were not available and, therefore, we only evaluated risk factors in relation to all-cause, but not breast cancer-specific, mortality even though some of the risk factors, such as BMI, are important predictors of death from other causes [54]. Nonetheless, the consistency of our results for all-cause mortality and for breast cancer recurrence indicate that these factors may contribute to breast cancer-specific mortality in a similar manner.

## Conclusions

In conclusion, our data indicate that risk factors for breast cancer are differentially associated with tumor subtypes and exert subtype-specific influence on survival/recurrence from the disease. Specifically, we observed that menarche after the age of 15 years, FFP after 30 years, underweight or overweight BMI, and breastfeeding practices were associated with survival/recurrence only among women with luminal A-like tumors. These findings are supportive of the prognostic value of reproductive and lifestyle-related factors in tumors with biologically favorable profiles, and could have implications for clinical counseling and for the development of subtype-specific prognostic tools. Future prospective studies are needed to delineate the role of lifestyle modification, especially changes in BMI, in improving clinical outcomes for women with luminal A-like breast cancer.

## Abbreviations

ACM: All-cause mortality
BMI: Body mass index
CI: Confidence interval
ER: Estrogen receptor
FFP: First full-term pregnancy
HER2: Human epidermal growth factor receptor 2
HR: Hazard ratio
IHC: Immunohistochemical
KM: Kaplan-Meier
LR: Likelihood ratio
OR: Odds ratio
PR: Progesterone receptor
